# Organochlorine Exposure and Incidence of Diabetes in a Cohort of Great Lakes Sport Fish Consumers

**DOI:** 10.1289/ehp.0800281

**Published:** 2009-03-06

**Authors:** Mary Turyk, Henry Anderson, Lynda Knobeloch, Pamela Imm, Victoria Persky

**Affiliations:** 1 Division of Epidemiology and Biostatistics, School of Public Health, University of Illinois–Chicago, Chicago, Illinois, USA; 2 Wisconsin Division of Public Health, Bureau of Environmental Health, Madison, Wisconsin, USA

**Keywords:** DDE, diabetes, dioxin, Great Lakes sport fish, PCB, prospective, sport fish

## Abstract

**Background:**

Studies have demonstrated ubiquitous human exposure to persistent organic pollutants (POPs) such as *p*,*p*′-diphenyldichloroethene (DDE) and polychlorinated biphenyls (PCBs). Although there is considerable evidence that POP exposures are associated with prevalent diabetes, these studies do not establish causality because the cross-sectional study design does not allow for assessment of temporality of the exposure–disease association. Prospective studies, however, have been lacking.

**Objectives:**

This study was designed to determine whether POP body burdens are related to incidence of diabetes in a cohort of Great Lakes sport fish consumers.

**Methods:**

The cohort was established in the early 1990s and followed through 2005. We tested serum for DDE and PCB congeners and assessed diabetes diagnosis, demographics, and fish consumption. Associations of diabetes with exposures were examined prospectively in participants without diabetes in 1994–1995, followed through 2005. Annual percent changes in DDE and PCB-132/153 from 1994 to 2005 were examined by diabetes status.

**Results:**

DDE exposure was associated with incident diabetes. Incident diabetes was not associated with mono-*ortho* PCB-118, total PCBs, or years of sport fish consumption. Annual percent change in DDE and PCB-132/153 did not differ significantly by diabetes status.

**Conclusions:**

This study demonstrates an association between DDE exposure and incident diabetes. The findings of an association of DDE with incident diabetes and the lack of effect of diabetes on annual percent change in POPs do not support the hypothesis that associations of POPs with diabetes are attributable to reverse causality. Additional studies should address the biological pathways by which DDE could affect glucose homeostasis.

A worldwide epidemic of diabetes is under way (Wild et al. 2004). Although obesity is a dominant risk factor, increased risk of diabetes is also related to sedentary lifestyle, poor diet, older age, ethnicity, family history of diabetes, hypertension, and dyslipidemia. Recently, a number of investigations have found associations of diabetes with persistent organic pollutants (POPs), such as polychlorinated biphenyls (PCBs), polychlorinated dibenzo-*p*-dioxins, and *p*,*p*′-diphenyldichloroethene (DDE), which are detectable in most of the U.S. population at relatively low levels [Centers for Disease Control and Prevention (CDC) 2005]. Diabetes has been associated with dioxin-like chemicals, non-dioxin-like PCBs, DDE, and/or other organochlorine pesticides in several cross-sectional investigations (Codru et al. 2007; Cox et al. 2007; Everett et al. 2007; Fierens et al. 2003; Lee et al. 2007b; Persky et al. 2002; Radikova et al. 2004; Rylander et al. 2005; Uemura et al. 2008).

Despite this increasing body of evidence, there is no clear evidence of a causal relationship between these exposures and diabetes because the cross-sectional study design does not allow for assessment of temporality of the exposure–disease association. The possibility that diabetes-related metabolic changes may affect POP metabolism, particularly with low-level exposures (Longnecker 2006), increases the importance of investigations using a cohort study design. Morgan et al. (1980) measured serum DDE + *p*,*p*′-dichlorodiphenyltrichloroethane (DDT) levels in 1971–1973 in workers exposed to pesticides and followed 3,669 workers to 1977–1978 for newly diagnosed diabetes. Workers with incident diabetes were found to have significantly higher DDE + DDT at enrollment compared with workers without diabetes. Vasiliu et al. (2006) studied 1,384 adults prospectively for 25 years and found that PCBs were associated with incident diabetes in women but not in men and remained associated when cases occurring during the first 15 years of follow-up were excluded, suggesting that reverse causality was an unlikely explanation for the relationship. Investigations of Operation Ranch Hand veterans, who were exposed to 2,3,7,8-tetra-chlorodibenzo-*p*-dioxin (TCDD) through the application of herbicides in Vietnam, found associations of TCDD exposure with incident diabetes (Henriksen et al. 1997). Furthermore, no evidence was found to support decreased dioxin metabolic rates in relation to diabetes in Vietnam veterans (Michalek et al. 2003).

In the present study we examined data from a large cohort of frequent and infrequent Great Lakes sport fish consumers collected during 1992–2005 (Anderson et al. 1996, 2008; Hanrahan et al. 1999b). DDE, but not dioxin-like mono-*ortho* PCBs or total PCBs (∑PCBs), was related to prevalent diabetes in this cohort in cross-sectional data from 2004–2005 (Turyk et al. 2009). In the present study, we used a cohort study design to investigate the associations of POP body burdens in 1994–1995 with incident diabetes from 1995 to 2005. We also examined the hypothesis that associations of POPs with diabetes are attributable to reverse causality—that is, that metabolic changes related to diabetes slowed POP metabolism resulting in differential metabolism rates by diabetes status, by calculating annual percent change in POPs between 1994 and 2005 and comparing rates in participants with and without diabetes.

## Methods

### Subject recruitment

**Figure 1:**
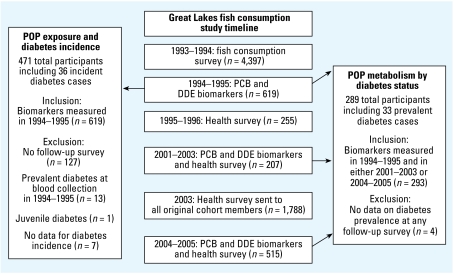
Design of the Great Lakes cohort study and analyses in this report. Arrows indicate source of exposure measurements for each analysis. Diabetes diagnosis and date of diagnosis for analyses was determined from follow-up surveys in 1995–1996, 2001–2003, 2003, and 2004–2005.

The Great Lakes Consortium for the Health Assessment of Great Lakes Sport Fish Consumption was organized in 1992 (Anderson et al. 1996). Figure 1 shows the time line for the study. Originally, > 4,200 participants with frequent and infrequent Great Lakes sport fish consumption were recruited and completed a telephone survey assessing demographic characteristics such as age, sex, height, weight, and fish consumption habits. The cohort included Great Lakes fishing charter boat captains, anglers who fished in inland Wisconsin lakes, and infrequent consumers (reporting consumption of fewer than six meals of Great Lakes sport fish in any year in the previous 20 years); spouses were included if the participant had had a child in 1970 or later. Based on the sport fish consumption reported in the survey, a stratified sample of 619 (520 frequent and 99 infrequent) Great Lakes sport fish consumers agreed to donate a blood specimen in 1994–1995 (Hanrahan et al. 1999b), and 209 participants donated blood again in 2001–2003. Health information was collected from some of these cohorts in 1995–1996 (Persky et al. 2001). Finally, health information was collected in 2003 from 1,788 of the initial 4,200 participants, and blood samples were collected in 2004–2005 from 515 these participants (Anderson et al. 2008). In total, 293 individuals from the original blood collection in 1994–1995 provided a second blood sample for analysis in 2001–2005 (Knobeloch et al. 2009). The study protocol was approved by institutional review boards at the University of Wisconsin–Madison and University of Illinois–Chicago, and all subjects gave written informed consent before participation.

### Health assessments

Because the study was originally designed as an exposure investigation, data on health conditions were not collected at baseline. Follow-up surveys in 1995–1996, 2001–2003, 2003, and 2004–2005 assessed self-reported diagnosis of diabetes, date of diagnosis, demographics, height, weight, smoking, alcohol use, medication use, and years of sport fish consumption.

### Biomarker analyses

Blood was collected in red-top Vacutainer tubes, allowed to clot for 20 min, centrifuged for 15 min, transferred to solvent-rinsed glass vials, and stored at –20°C until analysis. We analyzed samples for DDE and PCB congeners as previously described (Anderson et al. 2008; Hanrahan et. al. 1999b). We extracted sera with hexane/ethyl ether, with cleanup and fractionation using Florisil, silica gel, and concentrated sulfuric acid. PCB congeners and DDE were analyzed by gas chromatography. We monitored quality control with method blanks, spikes of bovine serum, duplicates of bovine serum spikes or sample duplicates, surrogate spikes, and confirmation of the analytes by second column or gas chromatography–mass spectrometry, as appropriate.

Total cholesterol and triglycerides were measured by Quest Diagnostics (Auburn Hills, MI, and Wood Dale, IL) in samples collected in 2004–2005 and by Meriter Laboratories (Madison, WI) in samples collected in 2001–2003. We calculated total serum lipids by the formula total lipid = [total cholesterol (mg/dL) × 2.27] + triglycerides (mg/dL) + 62.3.

### Statistical analyses

In this report, we present analyses of data from two subgroups of participants (Figure 1). First, we studied incident diabetes from 1995–2005 in participants who were without diagnosed diabetes at exposure assessment in 1994–1995. Second, we examined the effect of diabetes on rates of metabolism of DDE and PCB-132/153 in individuals who had repeat exposure measurements in 1994–1995 and 2001–2005.

We summed congener-specific values for selected PCBs (Knobeloch et al. 2009) to yield ∑PCBs (sum of PCB congeners 74, 99, 118, 146, 180, 194, 201, 206, 132/153, 138/163, 170/190, 182/187, and 196/20). We included PCB-118, a dioxin-like mono-*ortho* PCB congener, in the sum and also examined it separately (Van den Berg et al. 2006). Values for individual PCB congeners that were below the limit of detection (LOD) were imputed as zero. Analyses repeated with values below the LOD imputed as the LOD/2 for each specific congener yielded similar results (data not shown).

### Prospective analysis

In the group of participants with PCB and DDE (*n* = 619) measurements in 1994–1995, we identified incident diabetes through questionnaires administered in 1995–1996, 2001–2003, 2003, and 2004–2005 that assessed diabetes diagnosis and year of diagnosis. In total, 471 participants remained we excluded 127 without follow-up data, 13 with prevalent diabetes in 1994–1995, 1 with juvenile diabetes, and 7 with missing date of diabetes incidence (Figure 1).

We computed person-years of follow-up for each participant as the amount of time since the contaminants were measured in 1994–1995 to the date of the first diabetes diagnosis or the date of the last completed survey. We calculated incidence rates for diabetes (incidence/1,000 person-years) by dividing the number of cases of disease by the number of person-years of follow-up and multiplying by 1,000. We used the Cox proportional hazards regression model to evaluate the effect of exposure to POPs and sport fish consumption on diabetes incidence, using ordinal exposure variables or natural log transformations of continuous exposure variables. We obtained estimates of parameters and standard errors using PROC PHREG in SAS (version 9.1; SAS Institute Inc., Cary, NC). We retained relevant cofounders, including age, age squared, body mass index (BMI), sex, serum lipids, smoking, alcohol use, all fish meals in the last year, and Great Lakes sport-caught fish meals in the last year, in the models based on biologic and statistical criteria. Although serum lipids, smoking, and alcohol use were not assessed at baseline, we obtained estimates of these variables during the follow-up. Age, sex, and BMI were included in all adjusted models, and additional covariates were added individually to this adjusted model. Confounding was defined as a change of > 10% in the incidence rate ratio (IRR) for the exposure after control for the covariate. We investigated effect modification using variables indicating the product of the potential effect modifier with the exposure, and by stratification of regression models by the potential effect modifier. We performed a sensitivity analysis after exclusion of participants who reported incident diabetes < 6 years after the exposure measurements in order to reduce the potential that associations were influenced by reverse causality.

### Metabolism analysis

In this analysis we included the 293 participants who had repeat organochlorine measurements at 1994–1995 and 2001–2005; four were excluded because diabetes status was missing in all follow-up surveys (Figure 1). Michalek et al. (2003) estimated dioxin elimination rates based on a first-order kinetic assumption. However, in the present study a half-life for DDE and PCB congeners could not be calculated for all participants because not all participants had decreased POP levels at the later measurements in 2001–2005, perhaps due to continued sport fish consumption (Knobeloch et al. 2009). Therefore, we chose to examine the hypothesis that metabolic changes related to diabetes slowed POP metabolism using annual percent change in DDE or PCB-132/153 concentration (Knobeloch et al. 2009).

We calculated annual percent change in DDE and PCB-132/153 as [(follow-up – baseline concentration)/baseline concentration]/time interval in years × 100. Values for DDE and PCB-132/153 that were below the LOD were imputed as the LOD/2. Annual percent changes were log-transformed, after the addition of a constant to move the minimum value of the distribution > 1, to approximate a normal distribution. We compared annual percent change for DDE and PCB-132/153 in participants who reported diabetes at any time during the study with those who reported no diabetes using least squares geometric means and 95% confidence intervals (CIs) estimated by analysis of covariance. We adjusted estimates for age, sex, BMI at baseline, percent change in BMI, and log baseline exposure level. We repeated this analysis using DDE and PCB-132/153 half-lives calculated for participants who had a decrease in body burden at the 2001–2005 measurement (half-lives were calculated as described by Knobeloch et al. 2009), which yielded similar results to the analysis of annual percent change in DDE and PCB-132/153 (data not shown).

## Results

### Prospective analysis: POP exposure in 1994–1995 and incident diabetes from 1995 to 2005

Participants who were without diagnosed diabetes when they had exposure measurements in 1994–1995 were followed for incident diabetes for an average of 8.4 years. Follow-up data was not available for 127 participants. These participants were more likely to be female, were significantly younger, and had lower ∑PCB and DDE levels compared with those with follow-up data (data not shown). Table 1 shows the characteristics of the participants with follow-up.

A total of 36 cases of diabetes were reported after the initial exposure assessment, for an incidence rate of 9.1/1,000 person-years in the entire cohort, 6.5/1,000 person-years in women, and 11/1,000 person-years in men (data not shown). We calculated incidence rates for subgroups of the cohort: infrequent Great Lakes sport fish consumers, Wisconsin inland lake anglers, and Great Lakes captains who fished from Lake Michigan, Lake Huron, or Lake Erie. Incidence/1,000 person-years was 6.9 in infrequent Great Lakes consumers and was higher for sport fish consumers for all lakes (Michigan, 9.5; Huron, 10.6; Wisconsin anglers, 13.3) except Lake Huron (4.4).

Geometric mean DDE, PCB-118, and ∑PCBs, but not years of sport fish consumption, were higher in participants who subsequently developed diabetes (Table 1). However, in proportional hazard models adjusting for age, BMI, and sex, only DDE remained associated with incident diabetes (Table 2). Age confounded all diabetes–exposure associations, and BMI confounded the associations of diabetes with DDE and PCB-118 (data not shown). A one-tertile increase in DDE corresponded to an IRR of 2.01 (95% CI, 1.20–3.66; *p* = 0.008) (data not shown), and a 1-natural-log increase in DDE corresponded to an IRR of 1.52 (95% CI, 1.03–2.24; *p* = 0.04; data not shown). The association DDE with incident diabetes remained significant with further adjustment for smoking, alcohol use, serum triglycerides, serum cholesterol, fish meals in last year, Great Lakes sport fish meals in last year, years of sport fish consumption, PCB-118, and ∑PCBs (Table 2).

When the 15 participants who developed diabetes during the first 6 years of follow-up were excluded from the analysis, the association of DDE with incident diabetes remained significant (IRR for increase in 1 tertile = 2.37; 95% CI, 1.20–4.66; *p* = 0.01) (data not shown).

We found no synergistic and/or antagonistic effects among exposures or between BMI or age and exposures in models using multiplicative interaction terms for exposure, BMI, and age tertiles (data not shown). In analyses stratified by BMI, the IRR for a one-tertile increase in DDE was ≥ 1.7 in all BMI strata, although the number of diabetes cases in the two lower-BMI groups was small and the IRRs in did not reach significance (Table 3). We found similar results in age-stratified models (Table 3). In sex-stratified analyses, the associations of DDE with incident diabetes were of borderline significance (*p*-value > 0.05) (Table 4), but the association of DDE with diabetes reached significance in men after further adjustment for ∑PCB tertiles.

### POP metabolism rates and diabetes

We examined the hypothesis that associations of POPs with diabetes are attributable to reverse causality—that is, that metabolic changes related to diabetes slowed POP metabolism, resulting in differential metabolism rates by diabetes status. We estimated metabolism using annual percent change in DDE and PCB-132/153 in 289 participants who had repeat measurements of serum PCBs and DDE in 1994–1995 and 2001–2005 and known diabetes status. Least-squares geometric means of the annual percent change in DDE and PCB-132/153 were not significantly different in participants with and without diabetes, with or without adjustment for age, sex, BMI, percent change in BMI, and natural log baseline exposure (Table 5).

## Discussion

In this investigation we found an association of DDE exposure, but not exposure to dioxin-like PCB-118, ∑PCBs, or years of eating Great Lakes sport fish with incident diabetes in a cohort of sport fish consumers.

The results of the present study are consistent with the general finding that exposure to lipid soluble POPs is related to diabetes [reviewed by Carpenter (2008)]. Associations with a single chemical are difficult to interpret because of concomitant exposures to other POPs, although adjustment for multiple exposures may be imprecise because exposures can be strongly correlated due to similar exposure routes. Unmeasured exposures as well as the potentially beneficial effects of omega-3 fatty acids on diabetes (Nettleton et al. 2005) might confound associations of diabetes with POPs in populations with high fish consumption, although our results did not suggest that there is confounding by fish-related exposures.

We noted consistent, dose-related associations of DDE with incident diabetes. This is similar to our findings from cross-sectional analyses of the same cohort (Turyk et al. 2009). Other investigations have also found an association of DDE or DDT with diabetes (Codru et al. 2007; Cox et al. 2007; Everett et al. 2007; Lee et al. 2007b; Morgan et al. 1980; Radikova et al. 2004; Rignell-Hydbom et al. 2007; Rylander et al. 2005). Several investigations concluded that pesticide exposure was related to diabetes, but not all have noted associations with DDT application (Beard et al. 2003; Cox et al. 2007; Montgomery et al. 2008; Morgan et al. 1980).

Our failure to find associations with non-dioxin-like PCBs is also similar to our cross-sectional analyses (Turyk et al. 2009) but not to the findings of most other cross-sectional investigations (Codru et al. 2007; Fierens et al. 2003; Lee et al. 2007b; Persky et al. 2002; Rignell-Hydbom et al. 2007; Rylander et al. 2005). Our results also did not confirm the association of PCB exposure with incident diabetes in women found by Vasiliu et al. (2006), a cohort assembled because of accidental dietary exposure to polybrominated biphenyls rather than high Great Lakes sport fish consumption. Potential explanations for these divergent results include lower exposure levels, shorter follow-up time, and smaller number of participants in our cohort.

The lack of an association of diabetes with dioxin-like PCB-118 is also consistent with our cross-sectional data (Turyk et al. 2009). However, our estimate of dioxin-like exposure (Van den Berg et al. 2006) is limited by our measurement of a single dioxin-like mono-*ortho* PCB congener, possibly biasing the findings toward the null hypothesis. Several investigations have examined the possibility that dioxin-like exposure is related to increased diabetes. The evidence is more consistent for lower-level exposures (Everett et al. 2007; Fierens et al. 2003; Henriksen et al. 1997; Lee et al. 2007b; Longnecker and Michalek 2000; Uemura et al. 2008) than for higher exposures, particularly for high-level TCDD exposures in workers from facilities involved in the manufacture of TCDD-contaminated herbicides [reviewed by Longnecker and Daniels (2001)].

POP metabolism is affected by body fat and changes in body fat (Wolff et al. 2005). We adjusted models for BMI, although if BMI is in the causal pathway this adjustment may be inappropriate. In the present study, BMI did not modify the effect of DDE on incident diabetes, but effect modification by body weight was found in several cross-sectional studies (Cox et al. 2007; Lee et al. 2007a).

We did not find sex differences in the effects of POPs on diabetes, as noted in several other studies (Persky et al. 2002; Rylander et al. 2005; Vasiliu et al. 2006). In this cohort, PCBs, DDE, years of sport fish consumption, and age were higher in men than in women. Although POP body burdens can be decreased by lactation, the differences in PCB and DDE body burdens were explained primarily by years of sport fish consumption and age, respectively, and not by lactation (Hanrahan et al. 1999a).

The higher incidence of diabetes in the men (11/1,000 person years) than in women (6.5/1,000 person years) may be related to higher DDE body burdens in the men. Similarly, the higher diabetes incidence rates in three of the four sport-caught fish consumer groups compared with the infrequent Great Lakes sport fish consumer group could reflect the higher DDE body burdens in the anglers (Hanrahan et al. 1999b). However, the group with the highest DDE body burdens [Lake Michigan (Hanrahan et al. 1999b)] did not have the highest diabetes incidence. Factors that might be related to the low diabetes incidence rate in the Lake Huron anglers are not obvious. Between 1995 and 2005, crude and age-adjusted diabetes incidence rates for adults 18–79 years of age were 5.9 and 6.1 per 1,000, respectively (CDC 2007). Extrapolating the CDC rates to the present study, we would have expected approximately 23–24 incident diabetes cases in our study population, if the age distributions were similar.

The present investigation has a number of limitations. As in any observational study, the association between DDE and diabetes does not establish causality. This investigation was not originally designed as a health study, so some important risk factors for diabetes were not determined at baseline, including smoking, alcohol use, serum lipid measurements, physical activity, and family history of diabetes. However, smoking, alcohol use, and serum lipids were estimated from follow-up data, and did not confound the effect estimates for DDE. Henriksen et al. (1997) did not find substantial attenuation of the association of TCDD with incident diabetes after adjustment for triglycerides, nor did they see effect modification by triglycerides. We assessed diabetes by self-report, which could have resulted in underdiagnosis and/or misclassification of disease. In a cross-sectional sample from our cohort taken in 2004–2005 (Turyk et al. 2009), we tested sera for hemoglobin A1c, which has been used as an indication of undiagnosed diabetes (Bennett et al. 2007). In the cross-sectional sample, we found elevated hemoglobin A1c (> 6.3%) in 4% of the participants without diagnosed diabetes, and 92% of those reporting diabetes also reported diabetes medication use or had elevated hemoglobin A1c levels. These findings suggest that some underdiagnosis, but not misclassification, of diabetes is likely to also be present in the current study.

The size of the study sample with only 10 years of follow-up limited our ability to examine the concurrent effects of multiple POP exposures. For DDE tertiles, the large 95% CIs in part reflect the small number of cases in the lowest tertile, which was the reference category. However, we also found a significant association of diabetes with the natural log of DDE. Loss to follow-up for the incidence analysis was greater for females, younger participants and those with lower organochlorine body burdens, but because diabetes disease status of these participants is not known, it is difficult to speculate whether these losses bias the analysis.

The strengths of this study include the longitudinal collection of POP body burdens and survey data in a cohort with exposures near the general population of the United States (Anderson et al. 2008) and the measurement of multiple exposures. Metabolic changes related to diabetes could be present years before diagnosis, and results of prospective studies could be affected by reverse causation if the time between exposure measurement and diagnosis is short. However, in the present study, when we excluded participants who developed diabetes during the first 6 years of follow-up, the association of DDE with incident diabetes remained significant (*p* = 0.01), suggesting that reverse causality was an unlikely explanation for the relationship. Similarly, in the Vasiliu et al. study (2006), the association of PCBs with diabetes remained when cases occurring during the first 15 years of follow-up were excluded. Additionally, the analysis of annual percent change in DDE and PCB-132/153 suggests that diabetes did not affect metabolism rates of these chemicals. A similar investigation also found no evidence to support decreased TCDD metabolic rates in relation to diabetes in Operation Ranch Hand veterans (Michalek et al. 2003).

Several mechanisms through which POPs, predominantly dioxin-like chemicals, could affect diabetes incidence have been investigated, including hyperinsulinemia, antagonism of peroxisome proliferator-activated receptor-γ (PPAR-γ) expression, induction of tumor necrosis factor-α (TNF-α), auto-immunity, and alterations in steroid metabolism. Dioxin exposure has been related to hyperinsulinemia in persons without diabetes (Cranmer et al. 2000; Henriksen et al. 1997; Lee et al. 2007a). Dioxin suppressed PPAR-γ protein expression (Cimafranca et al. 2004), induced TNF-α secretion, and decreased glucose transport and lipoprotein lipase activity *in vitro* (Kern et al. 2002) and decreased glucose transporter activity in mice (Liu et al. 1995). Elevated levels of autoantibodies associated with diabetes have been found in PCB-exposed workers (Langer et al. 2002). Potential mechanisms for the effects of DDE on diabetes have not been well studied, but DDE is known to have antiandrogenic effects, whereas DDT and other metabolites of DDE are estrogenic (Agency for Toxic Substances and Disease Registry 2002). Prospective studies suggest that testosterone may differentially modulate risk of diabetes in men and women, whereas sex hormone–binding globulin decreases risk in both men and women (Ding et al. 2006). Further mechanistic studies are needed in animals and in humans, which should include biological measurements of risk factors for diabetes.

## Conclusion

The present study showing an increased incidence of diabetes among those exposed to higher levels of DDE at baseline, along with the observation that diabetes status did not affect annual percent change in DDE and PCB-132/153, confirms previous cross-sectional studies and suggests the possibility of a causal relationship. Future studies should address the biological mechanisms by which DDE may be affecting glucose homeostasis.

## Figures and Tables

**Table 1:** Baseline demographic and exposure characteristics of participants included in incidence study by diabetes status.

					Men		Women	
Characteristic	Measure	No. (total)	Diabetes	No diabetes	Diabetes	No diabetes	Diabetes	No diabetes
No. of participants			36^a^	435	25^a^	254	11^a^	181
Age (years)	Mean	471	52.2**	47.9	53.1	50.6	50.2**	44.1
BMI (kg/m^2^ )	Mean	471	33.2**	27.8	31.9**	29.1	36.3**	26.0
∑PCBs (ng/g wet weight)	Geometric mean	471	3.61*	2.36	4.48	3.35	2.22	1.44
PCB-118 (ng/g wet weight)	Geometric mean	471	0.26**	0.15	0.29*	0.18	0.20	0.13
DDE (ng/g wet weight)	Geometric mean	471	6.01**	3.20	6.89**	3.92	4.42*	2.41
Years eating sport fish	Mean	471	30.0	24.7	31.3	28.5	27.0	19.3
Fish meals in last year	Mean	471	53.9	44.2	61.3	47.4	36.9	39.7
Great Lakes sport fish meals last year	Mean	469	34.9	30.2	42.7	34.2	17.1	24.5
Triglycerides in 2000–2005 (mg/dL)^b^	Mean	265	225.6	171.8	241.6	180.7	197.6	159.9
Total cholesterol in 2000–2005 (mg/dL)^b^	Mean	265	191.4	199.8	200.7	194.9	175.1*	206.3
Cigarette smoking during study^c^	Percent	456	11.4	18.8	8.3	24.6	18.2	14.6
Alcohol > 1/month during study^c^	Percent	452	77.1	83.9	84.0	86.5	60.0	80.4

a All participants had developed diabetes after POP exposures were measured in 1994–1995.

b Lipids were measured in participants who also donated blood in 2001–2003 or 2004–2005.

c Smoking and alcohol use were assessed during follow-up surveys from 1996–2005.

* *p* < 0.05 and

** *p* < 0.01 , Student’s *t*-test.

**Table 2:** Associations of incident diabetes with POP body burdens and sport fish consumption measured in 1994–1995 in 471 participants.

Exposure/tertile (ng/g wet weight)	No. per tertile	Incident cases	Person-years of follow-up	Incidence/1,000 person-years (no.)	Crude IRR (95% CI)	*p*-Value for trend	Adjusted IRR^a^ (95% CI)	*p*-Value for tertiles	*p*-Value for trend
DDE									

< LOD to 2.2	153	2	1,325	1.5	1	< 0.0001	1		0.008
2.3–5.3	162	12	1,336	9.0	6.0 (1.4–26.9)		5.5 (1.2–25.1)	0.03	
5.4–49.2	156	22	1,286	17.1	11.5 (2.7–48.9)		7.1 (1.6–31.9)	0.01	

∑PCBs									

< LOD to 1.6	157	6	1,346	4.5	1	0.07	1		0.37
1.6–4.3	157	15	1,277	11.7	2.6 (1.0–6.8)		2.0 (0.7–5.3)	0.17	
4.3–29.8	157	15	1,325	11.3	2.5 (1.0–6.6)		1.8 (0.6–5.0)	0.27	

PCB-118									

< LOD	187	9	1,588	5.7	1	0.02	1		0.54
0.1–0.3	124	8	1,040	7.7	1.4 (0.5–3.5)		0.9 (0.4–2.4)	0.87	
0.3–4.6	160	19	1,320	14.4	2.6 (1.2–5.6)		1.3 (0.5–3.0)	0.60	

Years eating sport fish									

0–15	158	9	1,323	6.8	1	0.20	1		0.89
16–35	160	12	1,344	8.9	1.3 (0.5–3.1)		1.1 (0.4–2.5)	0.91	
36–65	153	15	1,281	11.7	1.7 (0.7–3.9)		0.9 (0.4–2.3)	0.90	

Association of diabetes with DDE in all participants remained significant after adjusting for smoking (*n* = 456), alcohol use (*n* = 452), and serum lipids (*n* = 265) measured during the follow-up period; Great Lakes sport fish meals in the last year, all fish meals in the last year; and all other exposures (∑PCBs, PCB-118, and years eating sport fish), individually and simultaneously.

a IRR from proportional hazards model adjusted for age, sex, and BMI.

**Table 3:** BMI- and age-stratified associations of incident diabetes with DDE body burdens measured in 1994–1995 in 471 participants.

Stratum	DDE tertile (ng/g wet weight)	No. per tertile	Incident cases (no.)	Person-years of follow-up	Incidence/1,000 person-years (no.)	Crude IRR per one-tertile increase (95% CI)	*p*-Value for trend	Adjusted IRR per one-tertile increase^a^ (95% CI)	*p*-Value for trend
BMI (kg/m^2^)									

17–25	< LOD to 2.2	64	0	580	0	3.5 (0.5–25.8)	0.26	3.0 (0.4–26.2)	0.33
	2.3–5.3	43	1	385	2.6				
	5.4–49.2	28	1	243	4.1				
25–29	< LOD to 2.2	48	2	364	5.5	1.6 (0.6–4.3)	0.31	1.7 (0.6–4.9)	0.30
	2.3–5.3	67	2	553	3.6				
	5.4–49.2	43	4	331	12.1				
29–48	< LOD to 2.2	41	0	381	0	2.2 (1.2–4.1)	0.01	2.2 (1.1–4.3)	0.02
	2.3–5.3	52	9	398	22.6				
	5.4–49.2	85	17	712	23.8				

Age (years)									

25–44	< LOD to 2.2	83	1	752	1.3	1.8 (0.5–6.0)	0.33	1.5 (0.5–5.0)	0.48
	2.3–5.3	48	2	398	5.0				
	5.4–49.2	26	1	240	4.2				
44–52	< LOD to 2.2	46	1	370	2.7	2.0 (0.9–4.5)	0.08	1.5 (0.7–3.3)	0.26
	2.3–5.3	60	5	459	10.9				
	5.4–49.2	51	6	396	15.2				
52–76	< LOD to 2.2	24	0	204	0	2.9 (1.2–7.1)	0.02	2.6 (1.0–6.3)	0.04
	2.3–5.3	54	5	480	10.4				
	5.4–49.2	79	15	650	23.1				

a IRR from proportional hazards model adjusted for age, sex, and BMI.

**Table 4:** Sex-stratified associations of incident diabetes with POP body burdens and sport fish consumption in measured in 1994–1995.

Exposure	Tertile (ng/g wet weight)	No. per tertile	Incident cases (no.)	Person-years of follow-up	Incidence/1,000 person-years (no.)	IRR Crude (95% CI)	*p*-Value for trend	Adjusted IRR^a^ (95% CI)	*p*-Value tertiles	*p*-Value for trend
Men (*n* = 279)										

DDE	< LOD to 2.2	72	1	582	1.7	1	0.01	1		0.06*
	2.3–5.3	94	9	735	12.2	7.2 (0.9–56.7)		6.8 (0.9–54.5)	0.07	
	5.4–49.2	113	15	949	15.8	9.2 (1.2–69.4)		7.3 (0.9–56.5)	0.06	
∑PCBs	< LOD to 1.6	62	3	475	8.4	1	0.49	1		0.72
	1.7–4.3	92	10	756	13.2	2.1 (0.6–7.5)		1.8 (0.5–6.5)	0.40	
	4.3–29.8	125	12	1036	11.6	1.8 (0.5–6.4)		1.5 (0.4–5.5)	0.55	
PCB-118	< LOD	102	5	821	6.1	1	0.21	1		0.67
	0.1–0.3	63	8	503	15.9	2.6 (0.9–8.0)		2.1 (0.7–6.7)	0.19	
	0.3–4.6	114	12	942	12.7	2.1 (0.7–5.9)		1.4 (0.5–4.2)	0.54	
Years eating sport fish	0–15	69	6	533	11.3	1	0.95	1		0.69
	16–35	94	8	774	10.3	0.9 (0.3–2.6)		0.8 (0.3–2.4)	0.70	
	36–65	116	11	959	11.5	1.0 (0.4–2.7)		0.8 (0.3–2.2)	0.67	

Women (*n* = 192)										

DDE	< LOD to 2.2	81	1	743	1.3	1	0.003	1		0.08
	2.3–5.3	68	3	602	5.0	3.7 (0.4–35.8)		3.9 (0.4–42.0)	0.26	
	5.4–21.8	43	7	337	20.8	15.7 (1.9–130)		7.5 (0.7–78.3)	0.09	
∑PCBs	< LOD to 1.6	95	3	872	3.4	1	0.14	1		0.40
	1.6–4.2	65	5	521	9.6	2.8 (0.7–11.6)		2.3 (0.5–11.4)	0.32	
	4.3–10.7	32	3	289	10.4	3.0 (0.6–14.8)		2.2 (0.4–13.2)	0.40	
PCB-118	< LOD	85	4	768	5.2	1	0.05	1		0.51
	0.1–0.3	61	0	537	0	0		0	—	
	0.3–1.1	46	7	377	18.6	3.5 (1.0–12.1)		1.1 (0.2–5.3)	0.88	
Years eating sport fish	0–15	89	3	789	3.8	1	0.13	1		0.95
	17–35	66	4	570	7.0	1.8 (0.4–8.2)		1.7 (0.4–8.4)	0.50	
	36–56	37	4	323	12.4	3.2 (0.7–14.3)		1.1 (0.2–6.7)	0.95	

a IRR from proportional hazards model adjusted for age and BMI.

* Significant after further adjusting for ∑PCB tertiles.

**Table 5:** Annual percent change in DDE and PCB-132/153 from 1994–1995 to 2001–2005 in participants with and without diabetes.

		Annual percent POP change^a^			
		Unadjusted		Adjusted^b^	
Exposure (ng/g wet weight)	Diabetes status	Geometric mean (95% CI)	*p*-Value	Geometric mean (95% CI)	*p*-Value
DDE	Diabetes	–4.0 (–4.5 to –3.5)	0.41	–4.1 (–4.6 to –3.7)	0.64
	No diabetes	–4.6 (–5.8 to –3.3)		–3.8 (–5.1 to –2.5)	
PCB-132/153	Diabetes	–3.1 (–3.5 to –2.5)	0.24	–3.1 (–3.4 to –2.6)	0.55
	No diabetes	–3.8 (–5.0 to –2.5)		–3.5 (–4.6 to –2.3)	

Total number with data = 289; 33 reported diabetes at any survey, and 256 did not have diabetes.

a Least-squares geometric mean and 95% CI from analysis of covariance models.

b Adjusted for sex, age at 1994–1995, BMI at 1994–1995, percent change in BMI, and log POP in 1994–1995. Interactions between covariates and diabetes were not significant, and are not included in the adjusted model.
